# Leptin-LepRb Expressed in Gastric Cancer Patients and Related to Cancer-Related Depression

**DOI:** 10.1155/2017/6482842

**Published:** 2017-02-20

**Authors:** Yunbao Pan, Fuling Zhou, Chenyan He, Lingyun Hui, Tianhe Huang, Yongchang Wei

**Affiliations:** ^1^Department of Laboratory Medicine, Zhongnan Hospital of Wuhan University, Wuhan, Hubei 430071, China; ^2^Department of Hematology, Zhongnan Hospital of Wuhan University, Wuhan, Hubei 430071, China; ^3^Department of Clinical Oncology, The First Affiliated Hospital, Xi'an Jiaotong University, Xi'an 710061, China; ^4^Department of Clinical Laboratory, The First Affiliated Hospital, Xi'an Jiaotong University, Xi'an 710061, China; ^5^Department of Radiation and Medical Oncology, Zhongnan Hospital of Wuhan University, Wuhan, Hubei 430071, China

## Abstract

Depression is the most common psychiatric disorder among cancer patients. Studies have not only highlighted that leptin and its receptor (LepRb) are independent poor prognostic factors in gastric cancer (GC) patients but also shown that the leptin-LepRb is necessary for antidepressant-like behaviors. In this study, we examined the serum and tissue leptin-LepRb expression in GC patients. Enzyme-linked immunosorbent assay showed that depressive GC patients had significantly higher serum leptin-LepRb than healthy donors. Leptin-LepRb levels in GC tissues were also significantly higher than in matched paracarcinoma tissues using real-time RT-PCR. Moreover, we observed that both serum and tissue leptin-LepRb were significantly higher in depressive GC patients than those in nondepressive GC patients. Further, the patients with high tumor stage tend to have higher leptin-LepRb mRNA levels than that with low tumor stage. Together, our findings suggest that leptin-LepRb plays an important role in the pathogenesis and depression in GC. Leptin-LepRb therefore could be a potential diagnostic marker and therapeutic target in GC patients with depression.

## 1. Background

Cancer is a difficult disease, affecting patients both physically and emotionally. Despite medical progress, cancer is often considered synonymous with pain, suffering, and death. However, cancer is not only a certain end but a permanent condition with delayed or late effects of the disease and concurrent psychological disorder [1]. Increased risk for psychiatric morbidity among cancer patients was reported [1, 2]. Recently, depression has received increasing attention in cancer patients. Although depression may be a part of the reaction to diagnosis, depression persists in many patients, creating difficulties in general management and therapy [3]. There is also accumulating evidence indicating that affective and somatic depressive symptoms may occur prior to the establishment of a cancer diagnosis [4]. In addition, depression prolonged hospital stays and decreased survival [5, 6].

Gastric cancer (GC) is the fourth most frequent malignancy worldwide, behind lung cancer, breast cancer, and colorectal cancer. In China, GC is the third cause of death from the cancer [7]. Depression is the most frequent psychiatric issue among cancer patients. Cancer patients are likely to have depressive symptoms after a diagnosis of cancer or during the clinical course of cancer [3]. Studies have demonstrated that overall 21% of gastrointestinal patients suffer from depression [8, 9]. Importantly, patients' satisfaction with life is associated with depression.

Leptin, a hormone, is primarily produced by adipose tissue and secreted into plasma [10] and could be detected in various tissues [11, 12]. Leptin was initially recognized to control food intake and energy balance [10] and functions via its receptor (LepRb) [13]. Clinical studies demonstrated that elevated serum leptin levels at delivery could eventually serve as a biological marker for the prediction of depressive symptoms [14], suggesting a regulation of leptin secretion in depressive illness. Moreover, leptin alters along the antidepressant therapy [15]. Polymorphisms in the leptin gene and decreased leptin are associated with responses to antidepressants [16]. These studies indicated a role of leptin in depressive symptomatology and antidepressant therapy. Signaling pathways, such as Akt and extracellular-regulated kinase (ERK) 1/2, were associated with the therapeutic efficacy of antidepressant efficacy [17, 18]. These pathways are also recruited by leptin via LepRb [19]. Collectively, these findings suggest a critical role of leptin-LepRb in depression and antidepressant therapy.

Leptin-LepRb also regulates cell growth, apoptosis, cell differentiation, migration, and invasion in many carcinomas [20, 21]. Its tumorigenic action is mediated by JAK/STAT, PI3K/PTEN/Akt/mTOR, Raf/MER/ERK pathways [20, 21]. Serum leptin has been detected in various cancers with conflicting results. Decreased leptin levels were demonstrated in gastrointestinal cancer patients [22, 23]. However, increased leptin levels were showed in cachexia gastrointestinal and breast cancer patients [24, 25]. However, there is no report regarding association between leptin and depression in GC patients. The aim of the present study was to investigate the serum and tissue leptin-LepRb levels in GC patients with depression and to examine the relationship between leptin-LepRb and clinical factors in these patients.

## 2. Materials and Methods

### 2.1. Patients

All patients were from the First Affiliated Hospitals of Xi'an Jiaotong University (Xi'an, Shaanxi, China) between August 2008 and April 2009. The study group consisted of 56 men and 28 women with GC and the control group consisted of 14 men and 7 women with depression and 17 men and 13 women health donors. Patients who did not receive preoperative chemoradiation treatment were selected for this study. Ethical approval was obtained from the Xi'an Jiaotong University Ethics and Scientific Committee and met international standards for informed consent. The exclusion criteria were uncontrolled infections, cognitive problems, organic or psychotic disorder, a Karnofsky score no more than 70, and antipsychotic or antioxidant treatment. Depression was measured using the Diagnostic and Statistical Manual of Mental Disorders, Fourth Edition (DSM-IV).

### 2.2. Enzyme-Linked Immunosorbent Assay (ELISA)

The serum leptin and LepRb were detected using ELISA kits (R & D Systems, Minneapolis, MN, USA) according to the manual. Briefly, A total of 50 *μ*L serum were added to ninety-six-well plate coated with antibody and incubation at 37°C for 2 hours and then exposed to substrate of enzyme reaction. Then, chromogenic reaction was permitted for 30 min and then terminates the reaction by adding termination liquid. The absorbance was examined by microplate reader at 450 nm wavelength.

### 2.3. Real-Time RT-PCR

RNA was extracted from GC tissues and paracarcinoma tissues using RNA extraction kit (QIAGEN, Venlo, The Netherlands) according to the manual. 1 *μ*g total RNA was exposed to reverse transcription by reverse transcription kit (TaKaRa, Otsu, Shiga, Japan). Real-time PCR were performed using SYBR Green PCR Master Mix (TaKaRa, Otsu, Shiga, Japan). *β*-Actin gene was served as an internal control. Quantifications of mRNA were performed using the 2^−ΔΔCt^ method. Primer sequences of leptin were as follows: forward 5′-CCTGACTGGTGCTATAGGCTGGA-3′; reverse 5′-GTGAGTGCGGTTTGACCACTG-3′. Primer sequences of LepRb gene were as follows: forward 5′-TCTTATGCTGGGATGTGCCTTAGAG-3′; reverse 5′-TGAATTTGGTGGCATGCAAGA-3′.

### 2.4. Immunohistochemical Analysis

The leptin and LepRb in the formalin-fixed, paraffin-embedded tissue were examined by immunohistochemical analysis. Briefly, the specimens were sectioned (4 *μ*m thick), mounted on Superfrost/Plus slides (Fisher Scientific, Pittsburgh, PA), and deparaffinized in two xylenes and rehydrated through graded alcohols to distilled water. The slides were incubated with the primary antibodies leptin (Abcam, ab16227, Hongkong) and LepRb (Abcam, ab104403, Hongkong) at 1 : 100 dilution overnight. The sections were incubated with secondary antibody kit (DAKO Corp) and the chromogenic substrate 3,3-diaminobenzidine tetrahydrochloride (DAB). Expression levels of leptin and LepRb were evaluated by online tool, ImmunoRatio (http://153.1.200.58:8080/immunoratio/), which calculates the percentage of positively stained area (labeling index) by using a color deconvolution algorithm for separating the staining components (diaminobenzidine and hematoxylin) and adaptive thresholding for area segmentation [26].

### 2.5. Statistical Analyses

Results were expressed as mean ± SD. Variance analysis and *t*-test were used to compare among groups by SPSS software package 17.0. Pearson's correlation analysis was used to determine the association between depression and tumor stage and association between serum leptin and tissue leptin levels. *P* < 0.05 was considered statistically significant.

## 3. Results

### 3.1. Patients' Clinical Characteristics

To analyze the serum leptin and LepRb concentration, we collected blood samples from 30 health donors (median age, 57; range, 29–79 years) and 21 depressive patients (median age, 54; range, 16–69 years) and 27 GC patients (median age, 59; range, 26–79 years). To further analyze the leptin and LepRb mRNA levels in GC, we extracted RNA from 84 GC tissues (median age, 61; range, 26–79 years) and 48 matched paracarcinoma tissues (median age, 59; range, 40–79 years).

### 3.2. Predictive Value of Leptin and LepRb for Depressive Gastric Cancer

**Figure 1:**
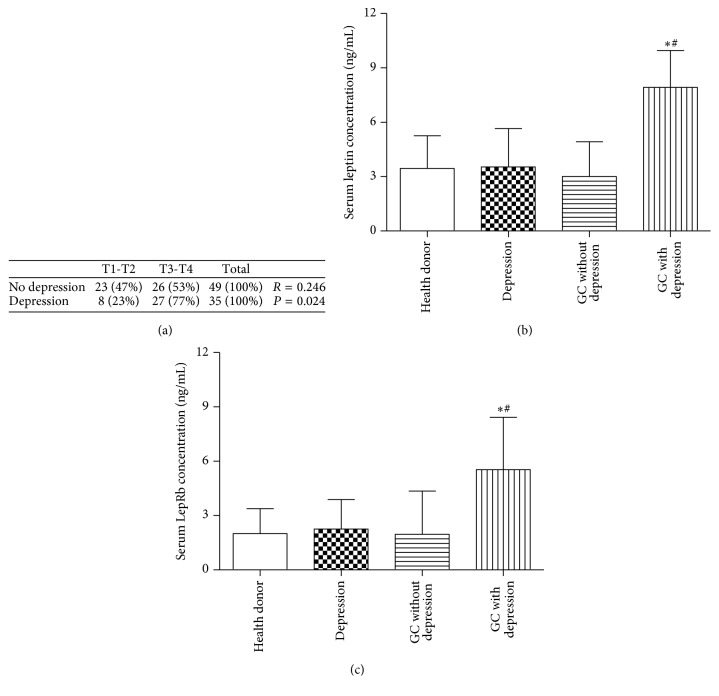
Serum leptin and LepRb levels in gastric cancer patients. (a) Depression was associated with tumor stage in GC patients. *R* values and *P* values from Pearson's correlation analysis. Leptin (b) and LepRb (c) levels in patients' serum were detected by ELISA. ^*∗*^Compared with health donor, ^*∗*^*P* < 0.05. ^#^Compared with the GC without depression, ^#^*P* < 0.05. GC: gastric cancer.

To elicit the relationship between depression and GC, we investigated the depression in 84 GC patients. The percent of patients in T3/T4 tumor stage in depressive cases was higher than that in nondepressive cases (77% versus 53%, *P* = 0.038, Fisher's exact test) (Figure 1(a)). Furthermore, depression was associated with high tumor stage in GC patients (*R* = 0.246, *P* = 0.024, Pearson's correlation analysis) (Figure 1(a)).

Expression levels of leptin and LepRb in serum were measured by ELISA. The leptin and LepRb levels were significantly higher in patients who diagnosed GC accompanied with depression (7.9 ng/mL and 5.5 ng/mL, resp.) than in either healthy controls (3.4 ng/mL and 2 ng/mL, resp.) or GC patients (3 ng/mL and 1.9 ng/mL, resp.). However, depression patients had the almost equal serum leptin and LepRb concentration (3.5 ng/mL and 2.3 ng/mL, resp.) with healthy donors (Figures 1(b) and 1(c)).

### 3.3. Discriminating Potential of Leptin and LepRb in Identifying Gastric Cancer Accompanied with Depression

**Figure 2:**
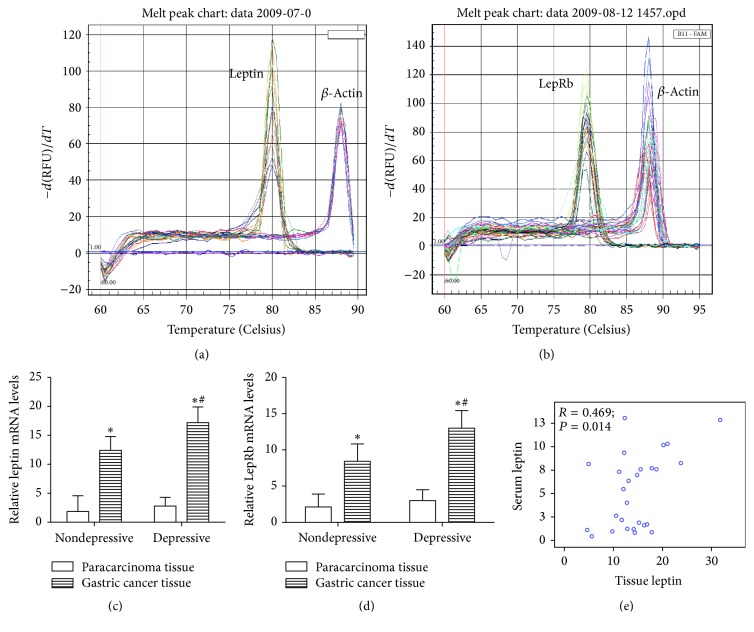
Tissue leptin and LepRb levels in gastric cancer patients. Leptin and LepRb levels in patients' tissue were detected by real-time RT-PCR. (a, b) PCR melting curve. (c, d) mRNA levels were indicated for leptin and LepRb. (e) Correlation of leptin in serum and cancer tissue at individual patients. ^*∗*^Compared with paracarcinoma tissue, ^*∗*^*P* < 0.05. ^#^Compared with nondepressive gastric cancer, ^#^*P* < 0.05.

To further study the leptin and LepRb expression in GC, we examined their mRNA expression in patients' tissues using real-time RT-PCR. PCR melting curve showed a single peak at ~80°C, 79°C, and 88.5°C from samples genes with leptin, LepRb, and *β*-actin, respectively, indicating specific amplification (Figures 2(a) and 2(b)). The leptin and LepRb mRNA levels were significantly increased in patients who diagnosed GC accompanied with depression compared with that of paracarcinoma tissues and GC without depression (depressive GC versus paracarcinoma tissues; 9.3-fold for leptin and 6.5-fold for LepRb, resp.; and depressive GC versus nondepressive GC, 1.4-fold for leptin and 1.4-fold for LepRb, resp.) (Figures 2(c) and 2(d)). Furthermore, we found that leptin in cancer tissue was associated with that in serum (*R* = 0.469; *P* = 0.014) (Figure 2(e)).

### 3.4. Leptin and LepRb Protein Expressed in Gastric Cancer

**Figure 3:**
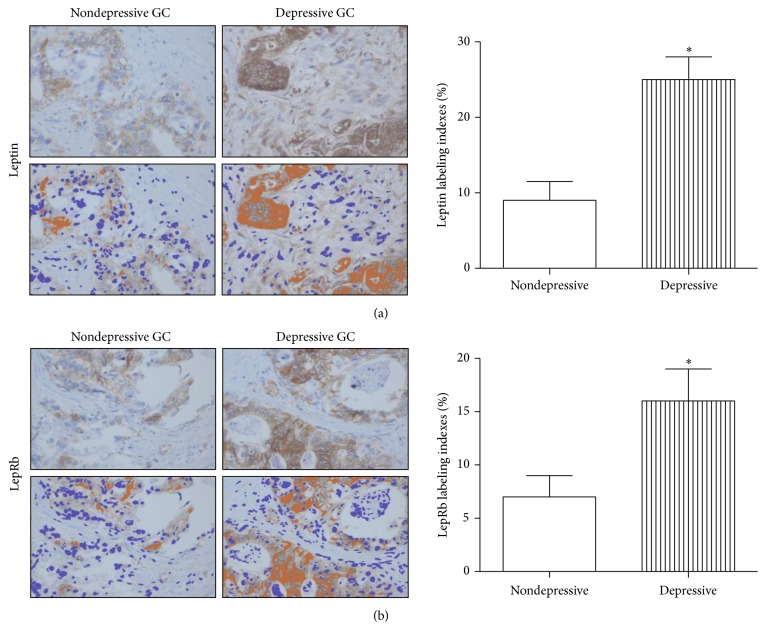
Immunohistochemical analysis of leptin (a) and LepRb (b) expression in nondepressive and depressive gastric cancer tissues. ImmunoRatio was used to evaluate the expression levels. (a) Original image; (b) pseudocolored image; ^*∗*^*P* < 0.05. GC: gastric cancer.

The protein levels of leptin and LepRb in GC tissues were detected by immunohistochemical analysis. The data demonstrated that leptin (Figure 3(a)) and LepRb (Figure 3(b)) protein levels were significantly increased (2.8-fold and 2.3-fold higher) in depressive GC patients (*n* = 16) compared with the nondepressive cancer patients (*n* = 16). These results were consistent with the leptin and LepRb mRNA levels identified in the GC tissues, indicating that leptin and LepRb may be important factors in depressive GC patients.

### 3.5. Correlation of Leptin and LepRb Expression with Clinic Characteristics

We assessed the mRNA levels of leptin and LepRb with various clinicopathologic variables in 84 GC patients. The patients with high tumor stage (III-IV) had higher leptin-LepRb mRNA levels than that with low tumor stage (I-II). A similar scenario has been obtained with respect to depression; depressive GC patients had higher leptin and LepRb mRNA levels than nondepressive patients. Our results, however, found no significant correlation between leptin-LepRb expression and age, sex, and lymph node metastasis (Table 1).

## 4. Discussion

As long-term cancer survivors increase, attention has been increasingly paid to cancer-related sequelae and their quality of life, including depression. Clinical depression was considered the most common psychiatric disorder among cancer patients [27]. Two reports using the DSM-III criteria found a 42% prevalence of depression [28] and a 33% prevalence in cancer patients [29]. It was reported that more than 20% disease-free breast cancer survivors continued to suffer from depression after completion of therapy. In our study, the DSM-IV scores of almost 41.7% of GC patients indicated depression. Stress appears to affect cancer progression [30]. Stress promotes inflammatory dysregulation and influences immune response in cancer models of depressive behavior [31]. Moreover, stress-induced autonomic response is associated with increased invasive potential [32, 33]. Additionally, stress promotes tumor cells resistance to apoptosis in the proinflammatory tumor microenvironment [34]. Our data indicated that depression was associated with high tumor stage in GC patients, suggesting a potential of depression in accelerating cancer progression.

Initially, leptin was thought to be expressed and secreted only by adipocytes; however, their production in gastric, colorectal, and mammary epithelial tissues has been documented [35–38]. Furthermore, leptin may act as a growth factor, participating in the development of cancer cell lines [39]. Serum leptin levels have been detected in various cancers patients with conflicting results [40–42]; however, the relation between psychiatric status and leptin-LepRb in GC patients has not been reported. In this study, we examined serum and tissue levels of leptin-LepRb in GC patients. In addition, we compared the serum and tissue leptin-LepRb levels between GC patients who suffer from depression and those without depression. We noted that GC patients had slightly lower serum leptin-LepRb levels than healthy donors, however, without statistical difference, and these data are consistent with other reports [25]. However, we observed significantly higher leptin-LepRb levels in GC tissue than in matched paracarcinoma tissue. Moreover, we observed that both serum and tissue leptin-LepRb were significantly higher in depressive GC patients than in nondepressive GC patients. This is the first study to examine the serum and tissue leptin-LepRb levels in GC patients with or without depression.

Although studies demonstrated a critical role of leptin-LepRb in depression and antidepressant therapy, several studies assessed leptin levels in the peripheral blood of people with bipolar disorder, with conflicting results. In mania and depression, some studies indicate that leptin levels are decreased, some are increased, and some show no difference when compared to healthy controls (reviewed in [43]). Recently, a meta-analysis on 1118 participants demonstrated that serum and plasma leptin levels were not altered in subjects with bipolar disorder when compared to healthy controls in mania, in depression, or in euthymia [43]. Our data are also consistent with this meta-analysis; we did not see difference between depression patients and healthy donors. Serum leptin levels seem to be higher in women than in men [44]. This gender-related difference could be explained by the fact that estrogen stimulates leptin production, whereas testosterone inhibits leptin production [45]. Similarly, our study showed that tissue leptin was slightly higher in women than in men in GC patients without statistical differences. This observation suggested that sex is not a determinant for leptin levels in cancer patients. Our data are also consistent with other studies [44] that age was not associated with leptin in GC patients.

Recently, studies highlighted that leptin-LepRb were independent poor prognostic factors in GC [46]. Leptin-LepRb were correlated with adverse clinicopathological parameters and inversely correlated with survival [46]. Similarly, our study showed that leptin-LepRb levels were higher in T3/T4 stage patients than those T1/T2 patients. As one of explanations for the fact that leptin-LepRb expression group had poor survival, researchers suggested that leptin-LepRb counteracted apoptosis in cancer cells [46]. Further studies are required to clarify the prognostic value of leptin-LepRb in GC.

## 5. Conclusions

In conclusion, we found that depressive GC patients have increased leptin-LepRb levels compared with nondepressive GC patients. The results from this study indicated that high tumor stage tends to have high serum and tissue leptin-LepRb levels.

## Figures and Tables

**Table 1:** Expression of leptin and leptin receptor in 84 gastric cancer patients.

Characteristics	Leptin expression	*P* value	LepRb expression	*P* value
Age (Y)				
<65	11.15 ± 2.38	>0.05	11.13 ± 2.63	>0.05
≥65	12.27 ± 1.68		10.33 ± 2.22	
Gender				
M	11.56 ± 1.65	>0.05	11.19 ± 2.76	>0.05
F	12.03 ± 2.04		12.21 ± 3.21	
Lymph node metastasis				
Negative	11.62 ± 2.92	>0.05	11.20 ± 2.67	>0.05
Positive	11.84 ± 2.75		11.39 ± 2.16	
Tumor stage				
I-II	9.02 ± 1.65	<0.05	8.37 ± 1.39	<0.05
III-IV	12.93 ± 2.43		12.80 ± 2.17	
Depression				
Positive	17.20 ± 2.71	<0.05	13.00 ± 2.45	<0.05
Negative	12.40 ± 2.41		8.45 ± 2.37	
